# A coculture system of *Epicoccum nigrum* with *Mucor racemosus* induces red pigment biosynthesis in vinegar mash

**DOI:** 10.1007/s00253-026-13956-1

**Published:** 2026-07-23

**Authors:** Fengli Wang, Fangyuan Xu, Xinle Liang, Wenjie Wang

**Affiliations:** College of Food Science and Biotechnology, Zhejiang Gongshang University, Hangzhou, Zhejiang 310018 People’s Republic of China

**Keywords:** Cocultivation, *Epicoccum nigrum*, *Mucor racemosus*, Pigment biosynthesis

## Abstract

**Abstract:**

Fungal pigments hold significant food industrial value due to their safe and sustainable properties. Here we identified a naturally occurring coculture pair *Epicoccum nigrum* SPDX618 and *Mucor racemosus* SPDX522 in vinegar mash that produced red pigments, which was not observed in either monoculture. Our results showed that *E. nigrum* acted as the pigment producer while *M. racemosus* served as the elicitor. Response surface methodology and pH stabilization strategy improved the yield of red pigment into 328.7 U/g. Through bioactivity-guided fractionation coupled with UHPLC-MS/MS and in silico structure reconstitution, four red pigments were preliminarily assigned as anthracycline-like compounds. RNA-seq analysis revealed a putative dual-PKS gene cluster (SPDX618009169–SPDX618009178) with exclusively upregulation in cocultured *E. nigrum* that is potentially involved in red pigment biosynthesis. Besides, the production of the yellow pigment epipyrone was reduced in cocultured *E. nigrum*, consistent with the downregulated transcription level of its biosynthesis genes. Comparative metabolomics analysis further revealed a broad downregulation in both of primary metabolism and secondary metabolism in coculture system, indicating that a competition interaction relationship also existed between *E. nigrum* and *M. racemosus*. Altogether, this study reports a fungal coculture system that exhibited red pigments production. These findings contribute to the understanding of microbial interaction-driven metabolite biosynthesis and may provide a foundation for future exploration of their potential application as a colorant.

**Key points:**

• *An Epicoccum nigrum–Mucor racemosus coculture system was identified to produce red pigment for the first time.*

• *E. nigrum acts as the red pigment producer and M. racemosus serves as the elicitor.*

•* RNA-seq analysis tentatively identified a putative dual-PKS gene cluster upregulated in coculture that is putatively involved in red pigment biosynthesis.*

**Supplementary Information:**

The online version contains supplementary material available at https://doi.org/10.1007/s00253-026-13956-1.

## Introduction

Fungi exhibit remarkable capacity for biosynthesizing structurally distinct secondary metabolites with multifaceted biotechnological applications, among which chromogenic compounds demonstrate exceptional commercial viability for sustainable production (Xu et al. 2023). Various pigment types, including melanins, anthraquinones, azaphilones, and carotenoids, have gained industrial importance in food coloration, agricultural applications, and pharmaceutical development (Kalra et al. 2020; Shen et al. 2023). To date, *Monascus* (Mapari et al. 2009), *Penicillium* (Sardaryan 2002), *Talaromyces* (Frisvad et al. 2013; Isbrandt et al. 2019), and *Blakeslea* (Liu et al. 2012) as well as their derived pigments, including monascorubrin, ankaflavin, mitorubrins, atrorosin and β-carotene, have achieved greatly industrial production by scaled-fermentation and have been authorized applications in food industry. Given the advances in metagenomics and culturomics from a variety of ecosystems including traditional fermented food microbiota, more and more fungi capable of producing pigment have been investigated. The filamentous fungus *Aspergillus ustus*, isolated from fermented broad bean paste, produces polyketide pigments, including pyrropyrazine diketone and dianthrone, which display attractive color and exhibit antimicrobial bioactivity (Zhou et al. 2023). The psychrotrophic polar fungus *Geomyces* sp. WNF-15A, isolated from Antarctic soil, synthesizes a water-soluble Antarctic Geomyces red pigment (AGRP) composed of polysaccharides, geomycamine, and derivatives, exhibiting a higher extinction coefficient than both commercial *Monascus* red pigment and cochineal red pigment (Long et al. 2024). The hydnoid fungus *Sarcodontia setosa* synthesizes novel quinone-type pigments setosic acid and 7,8-dehydrohomosarcodontic acid (Whaley et al. 2023). The exploration of novel pigment-producing fungi and pigments remains an active area of research.

Beyond well-established fermentation of sole species, cocultivation of multiple species has been extensively implemented in a variety of traditional food bioprocesses. Metabolic cross-talk within microbial consortia constitutes an indispensable biocatalytic driver in traditional fermented food ecosystems, critically determining textural, nutritional, and organoleptic properties of final products (Cheng et al. 2025; Nikoloudaki et al. 2024). Synergistic interactions encompassing bacterial-bacterial, bacterial-fungal, and fungal-fungal cross-talk are fundamentally involved in the fermentation ecosystems of traditional foods, which not only promotes quality and flavors but also benefits pigmentation to some extent. For example, red koji rice constitutes a symbiotic microbial consortium encompassing yeast cocultured with diverse strains of the genus *Monascus* capable of ethanol fermentation, pigmentation, and carbohydrate degradation activities (Moghadam et al. 2022).

In the context of synthetic microbiome design, the multispecies consortia that naturally evolved in traditional fermented foods are being reverse-engineered into modular and experimentally tractable model systems (Mohamed et al. 2022; Naz et al. 2026). These microbial networks provide a predictive blueprint for assembling resilient, functionally redundant fermentation communities and offer a low-risk alternative to innovation across the food industry. Zhejiang rosy vinegar is a traditional rice vinegar product, which is characterized by the natural surface fermentation over four months with a completely spontaneous complex microbiota and unique rosy appearance (Fang et al. 2023; Zhang et al. 2023). However, the source and the occurrence of its red pigment are still unclear. *Monascus* spp. have been isolated from this ecosystem, but *Monascus* red pigments are not detected in the final vinegar product and are not considered as the pigment signature (Tong et al. 2024).

In this study, we discovered a novel symbiotic association involving *Mucor racemosus* and *Epicoccum nigrum* from the traditional Zhejiang rosy vinegar mash that is capable of producing red pigments. In the coculture system, the pigment producer and the elicitor were identified, with the pigment structure tentatively characterized by tandem mass spectrometry complemented with SIRIUS computational prediction. Integrated analysis of transcriptome-metabolome interaction further revealed the regulatory network and symbiotic signaling molecules among two strains during the cocultivation. Our results deepen the understanding of *Epicoccum–Mucor* symbiosis and establish a framework for exploring the pigment biosynthetic gene cluster activated by interspecies interactions in fermented microbial consortia.

## Materials and methods

### Strains, media, cultivation conditions

*Mucor racemosus* SPDX522 (*Mr*S522) and *Epicoccum nigrum* SPDX618 (*En*S618) were isolated from traditional rosy vinegar in Zhejiang Province and stored at − 80 °C. Strains were first inoculated on potato dextrose agar (PDA) and incubated at 25 °C for 3 days, then punched with a cork borer to get 5-mm-diameter plugs. Two coculture configurations were then implemented: (i) 5-mm-diameter mycelial plugs pre-cultured from competing strains were positioned on opposing sides of 90-mm petri dishes with 3-cm separation (Oppong-Danquah et al. 2020); (ii) overlapping hyphal fragments (5 × 5 mm) from both strains were co-inoculated at the geometric center of the medium. Elucidating the red pigment producer strain in the coculture system was conducted as the previous description (Shang et al. 2017).

### Optimization of fermentation process for high-yield pigment production in coculture

The 5-mm-diameter plugs of *En*S618 and *Mr*S522 PDA inoculum were inoculated into 100 mL of Czapek-Dox rice medium at inoculation ratios of 1:1, 1:3, 1:6, and 1:9, followed by incubation at 25 °C with shaking at 180 rpm for 5 days. The red pigment yield was then determined. Pigment production was expressed as the absorbance at 505 nm (A₅₀₅) of the methanol extract per unit biomass, in units of U/g. The color value was calculated using the following formula:

$$Color\, value \,\left(U/g\right)=Dilution \,factor \,\times {A}_{505} \times 10/0.1$$

Subsequently, a one-factor-at-a-time experiment was conducted to screen the levels of each factor, followed by a Plackett–Burman (PB) design to identify key variables. The steepest ascent experiment was then employed to determine the central values of these factors. Finally, the optimal fermentation conditions were derived using Box–Behnken design (BBD) combined with response surface methodology (RSM). To address the issue of pH decline during fermentation, sodium acetate (NaAC) was supplemented at concentrations of 1, 3, 5, 10, 20, and 30 mmol/L under these optimized conditions for further testing.

### Large-scale fermentation, extraction and isolation of pigments

For large-scale fermentation, a total of 30 L of culture was performed using a batch fermentation in 150 flasks, containing 200 mL medium per 500 mL flask. The rice-based microbial biomass was harvested and ultrasonicated with methanol (1:10, w/v; 3 × 60 min cycles) using a 40-kHz probe sonicator. The resulting crude pigment extract was partitioned thrice with little petroleum ether in a separatory funnel to remove nonpolar impurities.

The crude pigment extract obtained was further processed in a silica gel column (150 cm) packed with spherical particle size 75–200 μm (Sigma-Aldrich) using a gradient elution of dichloromethane (CH₂Cl₂) and (MeOH, 10:1 to 1:1, v/v). Compound 8 was isolated from the C1 fraction (yellow pigment) by eluting the column with a 3:1 (v/v) solvent ratio. The C2 fraction (red pigment) obtained via 1:1 (v/v) elution was further purified by semi-preparative HPLC using a mobile phase of MeOH and 0.1% (v/v) formic acid (FA) with a linear gradient from 85 to 100% MeOH over 100 min at 5 mL/min, diluted fractions were collected, dried, and then stored at 4 °C for further analysis on UHPLC-Triple-TOF/MS and MS/MS.

### HPLC and UHPLC-Triple-TOF–MS/MS analysis

Chemical profiling of monoculture and coculture on rice medium was performed according to Shen et al. (2024). Chromatographic separation was achieved using a solvent system comprising MeOH (A) and water with 0.1% formic acid (B) at a flow rate of 0.6 mL/min, with a linear gradient from 10 to 100% A over 60 min. The UV absorption was monitored at 254 nm and 505 nm.

UHPLC-MS/MS analysis was performed following Oliveira et al. (2022) with modifications. Chromatographic separation was achieved on an ACQUITY UPLC T3 column (C18, 1.8 µm, 3.0 × 150 mm) at 45 °C with a mobile phase of 0.1% formic acid in water (A) and acetonitrile (B) at 0.35 mL/min. The gradient program was: 0 min 5%B; 15 min 50%B; 22 min 95%B until 25 min. MS and MS/MS data were acquired in IDA mode (± ESI) with source parameters: in the positive/negative mode, source voltage of 5.5 kV, the source temperature was 550 °C, collision energy (CE) was set at ± 40—± 20 V, the scan range of *m*/*z* of precursor ion and product ion were set as 80—1500 Da and 50—1500 Da. Controls (MeOH and blank medium) were analyzed identically. The structure of red pigments was predicted using SIRIUS 4 (Dührkop et al. 2019).

### Genome sequencing

The genome of *Mr*S522 and *En*S618 was sequenced by LingEn Biology (Shanghai, China) using Nova-seq 6000 (Illumina). The preliminary genome assembly was performed using SMRT Link v5.0.1 (Ardui et al. 2018; Reiner et al. 2018). Assembly completeness was evaluated with BUSCO v3.0.2, and coding genes were predicted using the Augustus 2.7 software. Functional annotation of genes was conducted through the Gene Ontology (GO) (Ashburner et al. 2000), Kyoto Encyclopedia of Genes and Genomes (KEGG) (Kanehisa and Goto 2000), Clusters of Orthologous Groups (KOG), Non-Redundant Protein Database (NR), and Swiss-Prot databases. The initial prediction and analysis of proposed biosynthetic gene clusters were carried out using antiSMASH (Blin et al. 2023) (http://antismash.secondarymetabolites.org/).

### Transcriptome sequencing and RT-qPCR

For RNA extraction, samples were prepared with monocultivation of *Mr*S522 and *En*S618 as well as cocultivation of two strains in 100 mL of Czapek and rice juice mixed liquid medium for 3 days at 25 °C. The cells were collected by centrifugation. Total RNA was extracted using TRIzol (Invitrogen) following previous method (Ma et al. 2026).

RNA-seq was performed by LingEn Biotech Co., Ltd. (Shanghai, China) using an Illumina NovaSeq 6000 platform. A dual RNA-seq strategy was adopted to analyze transcriptomic data as previously described (Westermann et al. 2012). Raw reads were first quality-trimmed using Trimmomatic (Bolger et al. 2014) for data preprocessing. The processed clean reads from monoculture were mapped to the reference genome of *En*S618 or *Mr*S522, respectively. And the clean reads from the coculture were separately aligned to the reference genomes of *En*S618 and *Mr*S522. The sequence alignment was performed using HISAT2 software (Kim et al. 2015), and stringent alignment thresholds were applied to intercept erroneous cross-species read mapping, taking full advantage of the abundant single-nucleotide polymorphisms (SNPs) and insertions/deletions (Indels) between two fungal genomes to eliminate interspecific mapping interference. Gene-level expression quantification was performed using featureCounts (v2.0.3) (Liao et al. 2014) with the parameters -M –primary. The -M option allows for the counting of multi-mapped reads, while –primary ensures that each multi-mapped read is assigned exclusively to its primary alignment site, which avoids redundant counting and reduces quantitative bias arising from ambiguous read mappings. The alignment quality was assessed using Qualimap (http://qualimap.bioinfo.cipf.es/) (Garcí­a-Alcalde et al. 2012), and differentially expressed genes (DEGs) were identified with edgeR under thresholds of |log₂FC|> 1 and FDR < 0.05. Functional enrichment of GO terms and KEGG pathways was analyzed using Goatools and KOBAS, respectively.

The reliability of species-specific transcript quantification results was further validated by quantitative qPCR using species-specific marker gene primers (Table S1). The qPCR reactions were performed using ChamQ™ Universal SYBR qPCR Master Mix (Vazyme).

### Untargeted metabolomics analysis

Untargeted metabolomic analysis was performed on monoculture and co-culture samples. Cellular extracts were analyzed using a QExactive™ HF Orbitrap mass spectrometer coupled to a Vanquish UHPLC system (Thermo Fisher Scientific), with analytical support provided by Lingen Biotechnology (Shanghai, China). Raw UHPLC-MS/MS data were processed in Compound Discoverer 3.1 for peak alignment, quantitation (parameters: retention time tolerance 0.2 min, mass tolerance 5 ppm, signal-to-noise ratio ≥ 3, minimum intensity 10^5^), and normalization to total spectral intensity. Molecular formulas were predicted based on adduct/fragment ion patterns. Metabolite identification was performed by matching peaks to mzCloud, mzVault, and MassList databases, followed by functional annotation via KEGG, HMDB, and LIPID Maps. Differential metabolite screening applied a *t*-test with thresholds of |log_2_(FoldChange)|> 1.0, *p* < 0.05, and VIP (variable importance in projection) ≥ 1.0 (Welling et al. 2021). Bioinformatic annotation of differentially accumulated metabolites (DAMs) was conducted using KEGG and HMDB databases. DAMs volcano plots were visualized using MetaboAnalyst (https://new.metaboanalyst.ca/home.xhtml) (Chong et al. 2018). DAMs lollipop plot and KEGG pathway enrichment analysis were performed via the ChiPlot (https://www.chiplot.online/.) (Ji et al. 2022). The metabolite-pathway interaction network was constructed using Cytoscape (Shannon et al. 2003).

### Statistical analysis

All data were derived from a minimum of three independent biological replicates and are expressed as mean ± standard deviation. Statistical significance was determined by one-way analysis of variance (ANOVA) followed by Duncan’s test using SPSS Statistics 24.0, with a significance threshold set at *p* < 0.05. All graphs were generated with OriginPro 2025 (OriginLab Corporation, Northampton, MA, USA).

## Results

### Identification of a unique *Epicoccum-Mucor* symbiosis capable of red pigment production

**Fig. 1:**
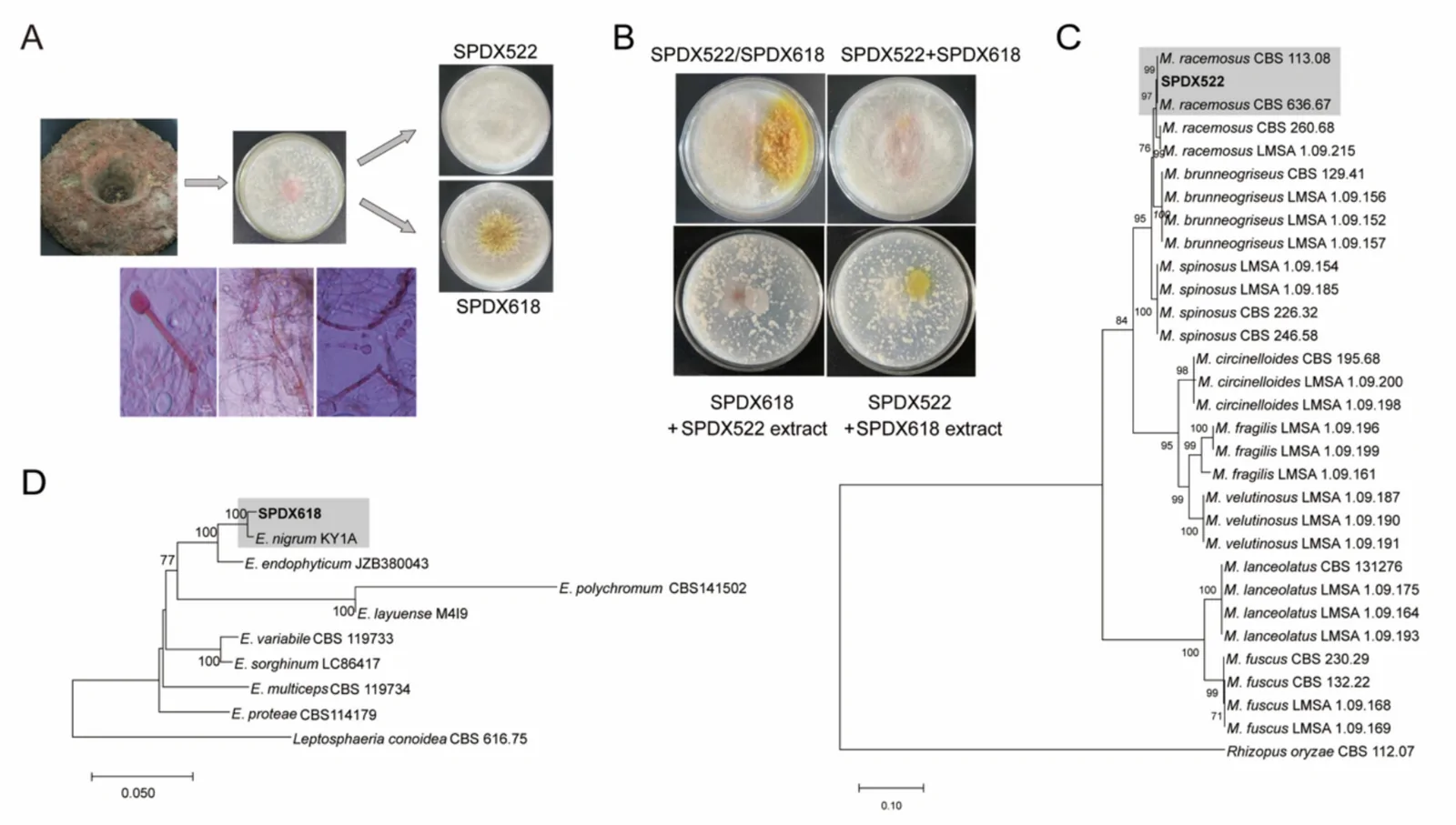
Isolation and identification of *En*S618-*Mr*S522 coculture capable of red pigment production from vinegar fermentation mash. (**A**) Isolation and purification of symbiotic red pigment-producing strains SPDX522 and SPDX618 from the vinegar mash. (**B**) Coculture systems: SPDX522/SPDX618 (separate inoculation), and SPDX522 + SPDX618 (mixture inoculation), SPDX618 + SPDX522 extract, SPDX522 + SPDX618 extract. (**C**) Phylogenetic tree of SPDX522 based on ITS-28S-Rpb2 joint sequences. (**D**) Phylogenetic tree of SPDX618 based on ITS-28S-Rpb2 joint sequences

Symbiotic fungal consortia exhibiting red pigmentation were selectively isolated from traditional rosy vinegar mash (Fig. 1A). Then two distinct strains were identified from the above plate and named as SPDX522 and SPDX618 (Fig. 1A). Out of our expectation, monoculture of either strain did not produce red pigment. SPDX522 showed no pigmentation on rice medium, while SPDX618 produced yellow pigments. But the red pigment produced again when cocultured SPDX522 and SPDX618, no matter separate or mixture inoculation in the same plate (Fig. 1B). To identify the producer of the red pigment, both strains were monocultured with the crude extract of each other. The results showed that SPDX618 produced red pigment with the crude extract of SPDX522, while SPDX522 could not produce red pigment with SPDX618 extract (Fig. 1B), indicating SPDX618 is the real producer of red pigment, and SPDX522 acts as the inducer.

Then the whole genome sequencing and the multi-locus sequence alignment, including the internal transcribed spacer (ITS) with intervening 5.8S nuclear ribosomal (nrDNA), partial 28S large subunit nrDNA (LSU) and RNA polymerase II second largest subunit (RPB2) regions, were conducted to elucidate the taxonomy of isolates SPDX522 and SPDX618. The phylogenetic trees based on ITS-28S-Rpb2 joint sequences revealed that isolate SPDX522 exhibited high similarity with *M. racemosus*, while isolate SPDX618 was classified into *E. nigrum* (Fig. 1C and D). So the isolate SPDX522 was renamed as *M. racemosus* SPDX522 (*Mr*S522), and the isolate SPDX618 was renamed as *E. nigrum* SPDX618 (*En*S618). According to the NCBI Taxonomy database, *Mr*S522 (accession number: SAMN54564409) belongs to the phylum Mucoromycota, while *En*S618 (accession number: SAMN54564408) belongs to the phylum Ascomycota, reflecting their broad phylogenetic divergence within the fungal kingdom (NCBI 2025). These two species are distantly related phylogenetically.

### Optimization of fermentation process for red pigment production

**Fig. 2:**
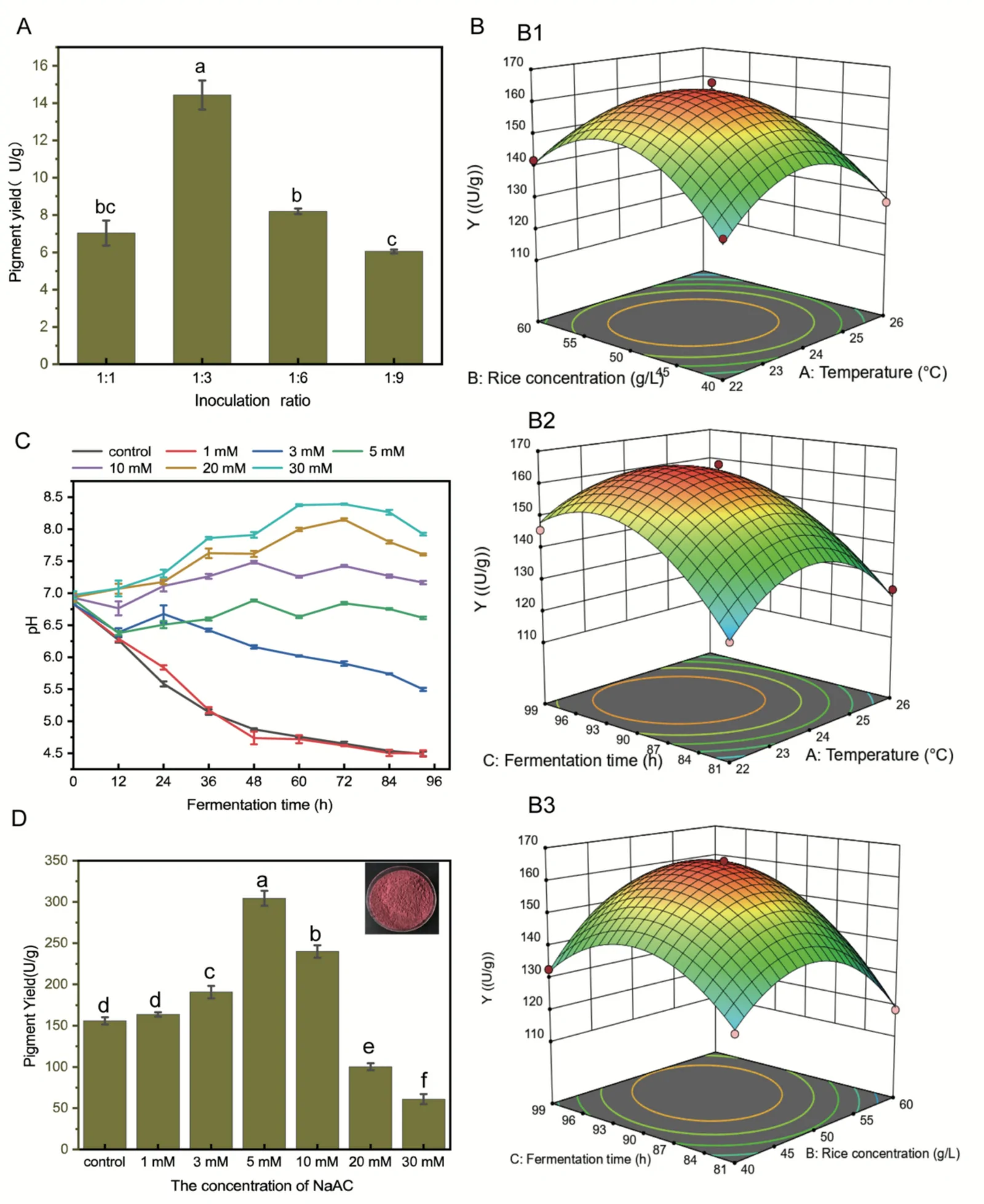
Optimization of fermentation conditions for red pigment production. (**A**) Effect of inoculation ratio of *En*S618 and *Mr*S522 on pigment production. (**B**) Response surface plots (B1/B2/B3) of the interaction effects for pigment yield. (**C**) Effect of different concentrations of sodium acetate on pH during fermentation process. (**D**) Effect of different concentrations of sodium acetate on pigment production and dried red rice powder from the coculture fermentation. Different letters above the columns indicate statistically significant differences between different groups. Values are the mean and standard deviation (SD) of three replicates

To establish an efficient red pigment production process, we first optimized the inoculation ratio of *En*S618 and *Mr*S522 in coculture system, determining a ratio of 1:3 (*En*S618:*Mr*S522) as optimal inoculation (Fig. 2A). This ratio likely achieved a balance between the inducer and producer, facilitating pigment production. Subsequently, single-factor experiment and Plackett–Burman design (PBD) were employed to screen key factors for red pigment production. The results showed temperature, rice concentration, and fermentation time were main factors, which further being optimized using Box-Behnken design and response surface methodology (Fig. S1, Tables S2–S6). The predicted optimal conditions are as follows (Fig. 2B): temperature 23.5 °C, fermentation time 92.8 h, and rice concentration 51 g/L, yielding a predicted pigment production of 163.5 U/g. Under these conditions, the pH decreased from 7.0 to 4.3 during fermentation (Fig. 2C). The addition of 5 mM sodium acetate stabilized the pH and further increased pigment production to 328.7 U/g (Fig. 2C, D).

### Preliminary characterization of red pigment in *EnS618-MrS522* coculture

**Fig. 3:**
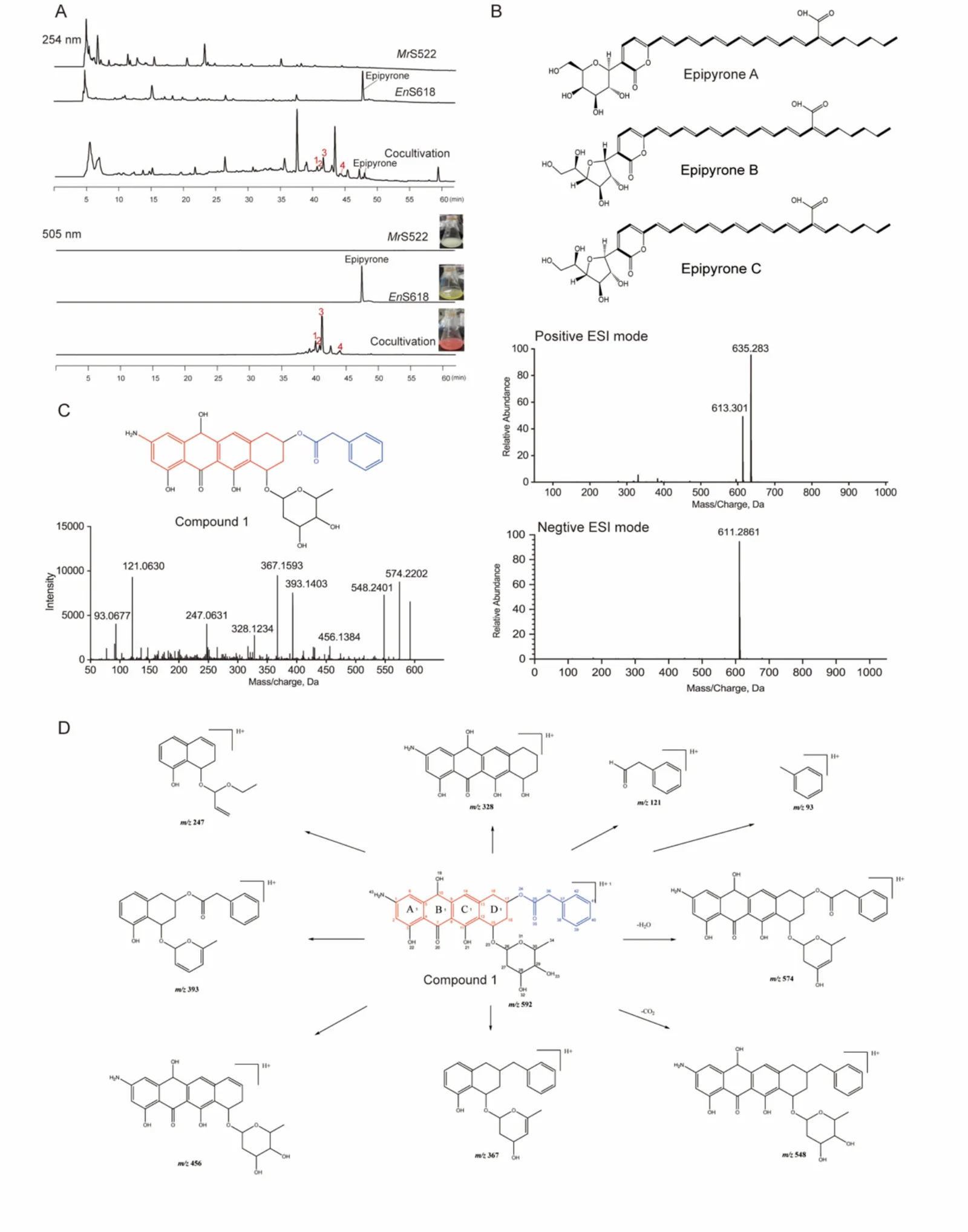
Preliminary characterization of pigments from *Mr*S522/*En*S618 coculture. (**A**) HPLC analyses of crude extracts from the monoculture and coculture. (**B**) Preliminary identification of Epipyrone with UHPLC-ESI-TOF–MS data. (**C**) Preliminary identification of compound (1) with MS/MS data. (**D**) Structural elucidation process of compound (1) using SIRIUS 4

To characterize the red pigment produced from the coculture, metabolites from monoculture of *En*S618 or *Mr*S522 and coculture were extracted and underwent LC–MS analysis at 254 nm and 505 nm (Fig. 3A). The monoculture of *En*S618 exhibited only one peak (47.9 min) at 505 nm. This peak was preliminary corresponded to epipyrone (C_34_H_44_O_10_) with *m*/*z* 613.301 ([M + H]^+^) and *m*/*z* 635.283 ([M + Na]^+^) in positive mode and *m*/*z* 611.286 ([M − H]^−^) in negative mode (Fig. 3B). Interestingly, the production of epipyrone decreased in coculture when compared to the monoculture of *En*S618.

Besides, more interests are focused on red pigment. Compared to the monoculture, coculture showed extra peaks after RT 35 min. A scale-up coculture fermentation with a total volume of 30 L was carried out using 150 flasks, and extensive efforts were devoted to purifying red pigments. But unfortunately, it is too hard to get enough pure compound for structure characterization. Here is some information of the red pigments that we obtained.

The red pigments (1–4) exhibited similar UV–Vis absorption lengths at 298–299 nm, 390–398 nm, and 484–507 nm (Table 1 and Fig. S2). They were tentatively characterized as anthracycline-like candidates through UHPLC-MS/MS and SIRIUS 4 analysis. Compound (1) exhibited hypothetical molecular ion signals at *m*/*z* 592.2155 ([M + H]^+^) and 590.2033 ([M − H]^−^) in positive and negative modes, respectively (Table 1, Fig. S3), with a proposed molecular formula of C_32_H_33_NO_10_. The predominant MS^2^ fragment ions at m/z 574, 548, and 456 correspond to neutral losses of H₂O, CO₂, and phenylacetic acid moiety (C₈H₇O₂), respectively (Fig. 3C). Besides, SIRIUS4 computed that Compound (1) might contain a similar core structure of a tetracyclic anthraquinonoid. The C-10 keto group on ring B is oxidized to a hydroxyl group, and the C-15 and C-17 on the D-ring are connected to a pyran structure and a phenylacetic acid group, respectively (Fig. 3D).

**Table 1:** The combined information of chromatographic, UV–Visible, and mass spectroscopy of red pigments

Compound	t_R_ (min)	λ_max_ (nm)	[M + H]^+^ (*m*/*z*)	[M-H]^−^ (*m*/*z*)	MS^2^ *(m*/*z*)
1	40.47	507, 391, 299	592	590	574, 548, 456, 393, 367, 247
2	41.02	484, 395, 299	528	526	510, 484, 393, 367, 247
3	41.35	502, 390, 298	542	540	524, 498, 406, 393, 367, 247
4	44.25	505, 398, 298	576	574	558, 532, 440, 393, 367, 247

Compound (2) exhibited hypothetical molecular ion signals at *m*/*z* 528.2216 ([M + H]^+^) and 526.2084 ([M − H]^−^) in positive and negative modes, respectively (Table 1, Fig. S4), with a proposed molecular formula of C_28_H_33_NO_9_. Compound (2) lacks the B-ring structure that present in Compound (1) but retains homology in its major cleavage fragment patterns (Fig. S4).

Compound (3) exhibited hypothetical molecular ion signals at *m*/*z* 542.2361 ([M + H]^+^) and 540.2243 ([M − H]^−^) in positive and negative modes, respectively (Table 1, Fig. S5), with a proposed molecular formula of C_29_H_35_NO_9_. Structural analysis revealed that Compound (3), while sharing a core scaffold with Compound (2), incorporates a methyl substitution at the C-1 unsaturated bond (Fig. S5 and S6).

Compound (4) exhibited hypothetical molecular ion signals at *m*/*z* 576.2205 ([M + H]^+^) and 574.2901 ([M − H]^−^) in positive and negative modes, respectively (Table 1, Fig. S7), with a proposed molecular formula of C_32_H_33_NO_9_. Compound (4) lacks a hydroxyl group at the C-10 position relative to Compound (1), as evidenced by comparative MS^2^ fragmentation profiles (Fig. S7 and S8).

### Transcriptome analysis revealed an upregulated polyketide gene cluster in the cocultured* E. nigrum* which might be response for red pigment biosynthesis

**Fig. 4:**
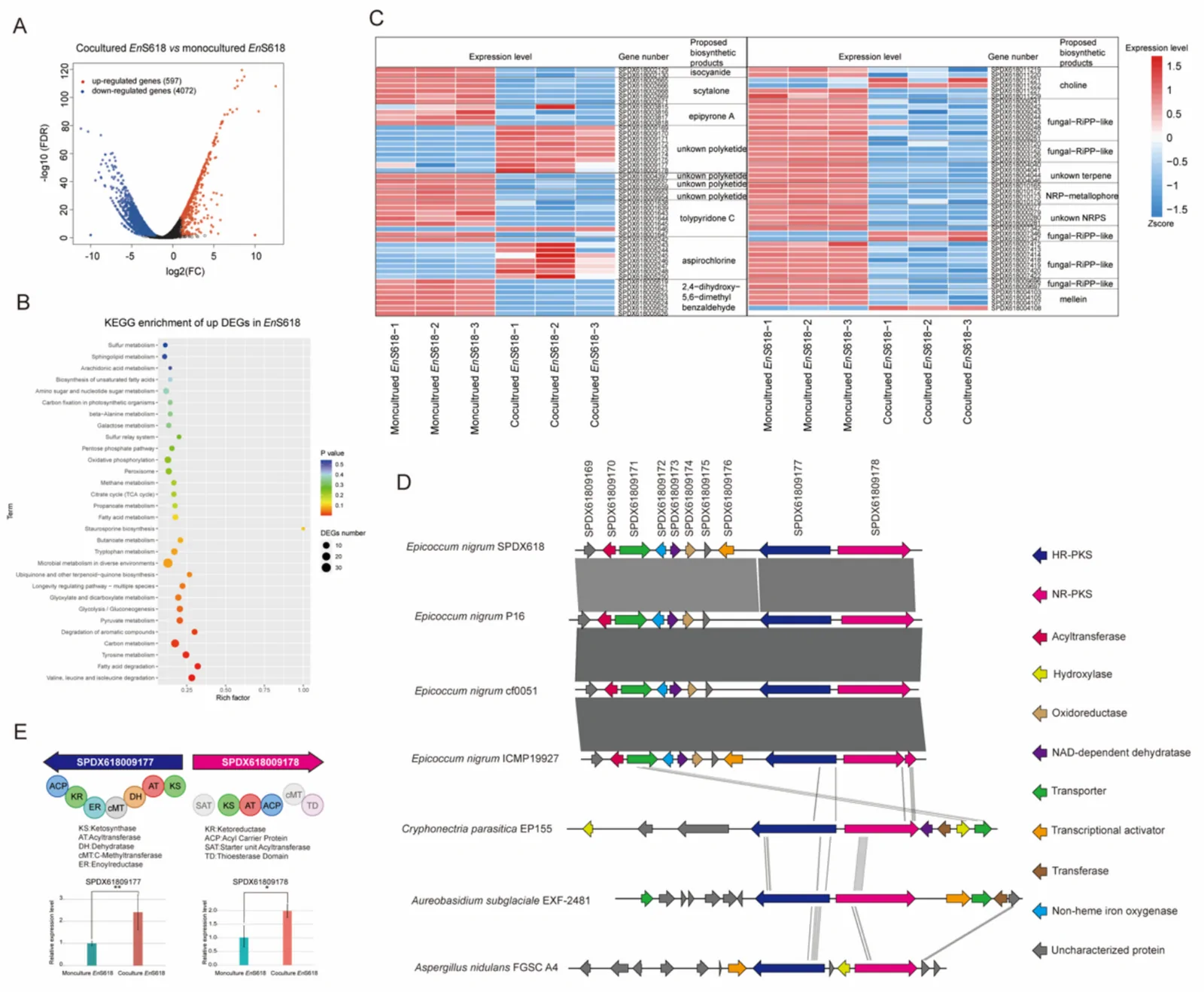
Identification of a putative dual-PKS gene cluster that might be responsible for red pigment biosynthesis. (**A**) Differentially expressed genes (DEGs) in cocultured *En*S618 when compared with monoculture. (**B**) KEGG pathway enrichment analysis of upregulated DEGs in cocultured *En*S618. (**C**) Expression level of 20 up- or down- regulated BGCs in cocultured *En*S618. (**D**) Alignment of homologous dual-PKS gene cluster in *E*. *nigrum* SPDX618 with other *E*. *nigrum* isolates and across fungal kingdom. (**E**) Domain architecture and RT-*q*PCR results of two PKS genes (*SPDX618009177* and *SPDX618009178*)

To obtain the putative biosynthetic gene cluster (BGC) that related to red pigment, RNA-seq was conducted from monocultured and cocultured *En*S618. The expression level of each transcript was calculated by the fragments per kilobase of exon per million fragments mapped (FPKM), and 597 genes were upregulated among 11,288 detected genes filtered by |log_2_FC|> 1 and FDR < 0.05 (Fig. 4A). Further KEGG analysis demonstrated that most upregulated genes were associated with lipid metabolism (fatty acid degradation, unsaturated fatty acid biosynthesis, arachidonic acid, and sphingolipid metabolism), carbohydrate metabolism (pyruvate metabolism, glycolysis/gluconeogenesis, glyoxylate/dicarboxylate metabolism, TCA cycle, pentose phosphate pathway, galactose, and amino/nucleotide sugar metabolism), amino acid metabolism (valine/leucine/isoleucine degradation, tyrosine/tryptophan metabolism) and energy metabolism (Fig. 4B).

Besides, the transcription level of 45 pre bedicted BGCs in *En*S618 was analyzed in monoculture and coculture. The that results showed 17 BGCs were downregulated and 3 BGCs got upregulated in the cocultured *En*S618 when compared to monoculture (Fig. 4C). Among the upregulated BGCs, one is a nonribosomal peptide synthase (NRPS) gene cluster (*SPDX618005243*-*SPDX618005251*) predicted to responsible for aspirochlorine biosynthesis, which is an antibiotic functions as a fungal protein inhibitor (Sakata et al. 2014). Another is a fungal ribosomally synthesized and post-translationally modified peptide (RiPP)-like cluster (*SPDX618007346*_*SPDX618007347*). The last is an unknown polyketide synthase (PKS) gene cluster (*SPDX618009169*-*SPDX618009178*) with 10 genes. This cluster contains two PKS genes (*SPDX618009177* and *SPDX618009178*), an acetyltransferase gene (*SPDX618009170*), a transporter gene (*SPDX618009171*), a non heme iron oxidase gene (*SPDX618009172*), a NAD dependent dehydratase gene (*SPDX618009173*), a quinone oxidoreductase gene (*SPDX618009174*), a zinc binuclear (Zn(II)_2_Cys_6_) transcription factor gene (*SPDX618009176*) and two hypothetical protein genes (*SPDX618009169* and *SPDX618009175*) (Fig. 4D). Interestingly, this PKS cluster is quite conserved among *E. nigrum* isolates, but low conservation was exhibited across species.

The PKS gene *SPDX618009177* is a highly reducing polyketide synthase (HR-PKS) gene with KS/AT/DH/MT/ER/KR/ACP domains, while another PKS gene *SPDX618009178* is a non-reducing polyketide synthase (NR-PKS) gene with SAT/KS/AT/ACP/MT/TD domains (Fig. 4E). RT-*q*PCR results confirmed that both PKS genes (*SPDX618009177* and *SPDX618009178*) were upregulated in cocultured *En*S618 (Fig. 4E).

Contrast to the rarely upregulated BGCs in cocultured *En*S618, much more BGCs were downregulated (Fig. 4C), including the putative epipyrone cluster (*SPDX618003815*-*SPDX618003818*), which is consistent with the result of reduced epipyrone production in Fig. 3.

### Transcriptomic differences in *M. racemosus* during coculture

**Fig. 5:**
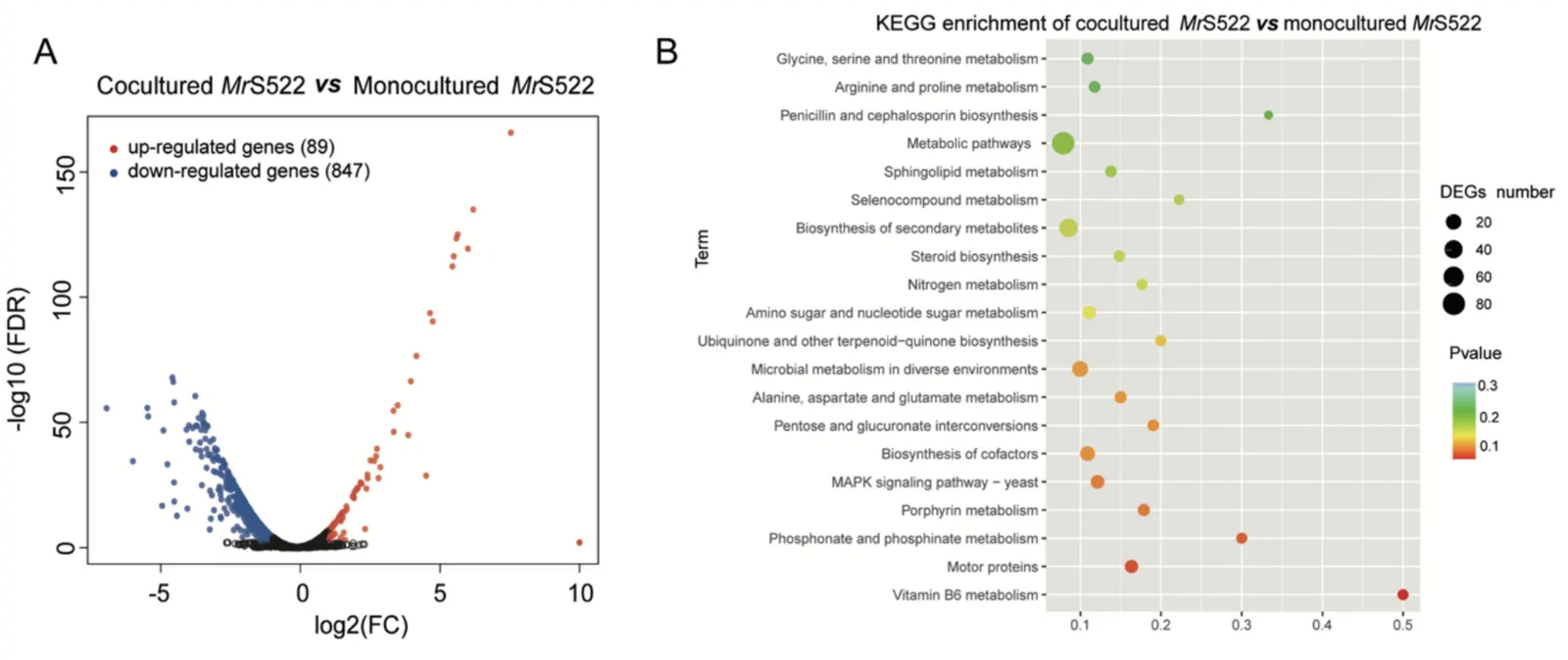
Transcriptomic differences in cocultured *Mr*S522. (**A**) Differentially expressed genes (DEGs) in cocultured *Mr*S522 when compared with monoculture. (**B**) KEGG pathway enrichment analysis of upregulated DEGs in cocultured *Mr*S522

The RNA-seq and transcriptomic analysis was also conducted to compare cocultured *Mr*S522 with monoculture. A total of 936 DEGs (9.1%) were detected in the cocultured *Mr*S522, including 89 upregulated genes and 847 downregulated genes (Fig. 5A). The most upregulated genes were NAD(P)H oxidoreductase genes (*SPDX522A06359* and *SPDX522A09791*), transporter genes (*SPDX522A05170* and *SPDX522A02494*), carotenoid isomerization/oxygenation gene (*SPDX522A06145*), and cytochrome P450-mediated oxidation gene (*SPDX522A04855*), while the most downregulated gene was a hyphal formation-regulating Homeobox transcription factor gene (*SPDX522A04085*), indicating the limitation of hyphal development and proliferation in *M*rS522 during coculture.

KEGG analysis further revealed significant reduction in vitamin B6 metabolism, motor proteins, phosphonate and phosphinate metabolism, porphyrin metabolism, and the MAPK signaling pathway (Fig. 5B, *p* < 0.05). But glutathione metabolism-related gene SPDX522A04300 (glutathione S-transferase, GST) and melatonin hydroxylation gene SPDX522A01110 (cytochrome P450, CYP450; log_2_FC > 1.0) were markedly upregulated, indicating an enhanced detoxification of ROS during coculture through glutathione conjugation and melatonin-derived antioxidant pathways.

### Comparative metabolomics analysis revealved metabolic alteration in coculture

**Fig. 6:**
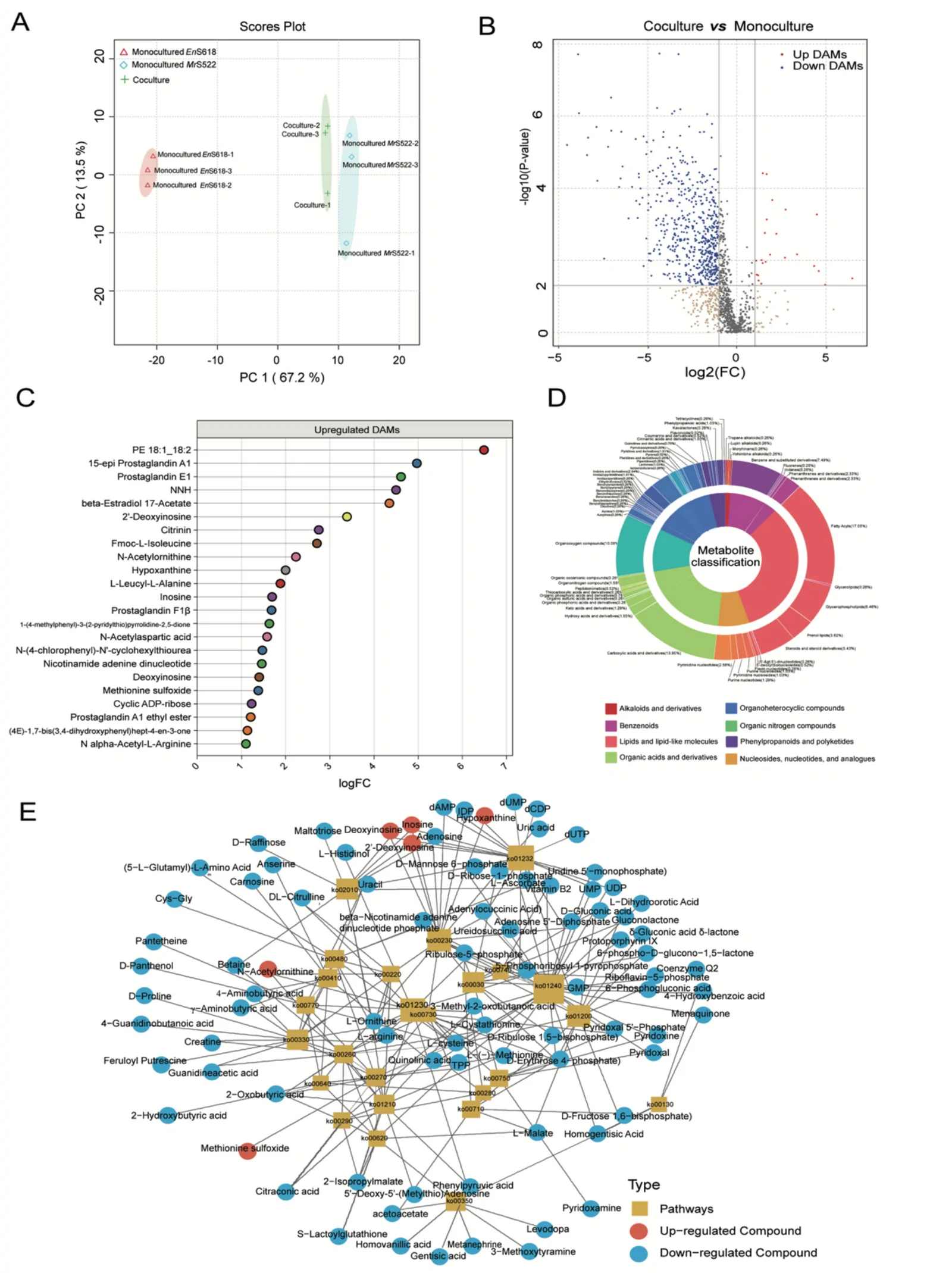
Comparative metabolomics analysis unravels key metabolic alterations in coculture. (**A**) Principal component analysis (PCA) of metabolic profiles of *En*S618 monoculture, *Mr*S522 monoculture, coculture and the sum of monoculture group (*En*S618 + *Mr*S522). (**B**) Different accumulated metabolites (DAMs) in coculture when compared with the sum of monoculture group. (**C**) The lollipop plot of upregulated metabolites in coculture. (**D**) Primary (inner circle) and secondary (outer circle) classification of downregulated metabolites. (**E**) Regulatory network mapping of DAMs

To comprehensively understand the metabolic alterations during cocultivation, comparative metabolomics analysis was then conducted to identify different accumulated metabolites (DAMs) in coculture when compared to monoculture groups. As shown in Fig. 6A, principal component analysis (PCA) scores plot showed distinct distribution between coculture and monocultures, indicating that a synergistic or antagonistic interaction occurred between *En*S618 and *Mr*S522. Besides, coculture was clustered with monocultured *Mr*S522, indicating a repression of *En*S618 during coculture interaction. The PCA loadings plot was generated and inspected using average data from replicates to evaluate which compounds contributed to the separation between groups. The results showed that 23 metabolites were upregulated and 587 metabolites were downregulated in coculture (|log_2_FC|> 1, *p* < 0.05), which reflects an inhibited metabolome mainly in *En*S618 (Fig. 6B).

A large amount of lipids decreased in coculture, but phosphatidylethanolamine (PE) and oxylipins prostaglandin derivatives such as 15-epi prostaglandin A1, prostaglandin E1 (PGE1), prostaglandin F1β (PGF1β), and prostaglandin A1 ethyl ester increased significantly (*p* < 0.05) when compared to monoculture (Fig. 6C and D). Furthermore, the elevated levels of methionine sulfoxide and derivatives of hypoxanthine and nucleoside (inosine, 2′-deoxyinosine, deoxyinosine) were observed. KEGG analysis showed a crash of pentose phosphate pathway (PPP), which might disrupt redox homeostasis and exacerbate intracellular oxidative stress (Fig. S9).

The reductions of various secondary metabolites were also observed in metabolome (Fig. 6D), including alkaloids and derivatives, benzenoids, phenylpropanoids and polyketides, which were consistent with our RNA-seq data in Fig. 4. Then a regulatory network analysis was conducted and 25 core metabolic pathways were identified, including the biosynthesis of cofactor (ko01240), nucleotide metabolism (ko01232), biosynthesis of amino acids (ko01230), 2-oxocarboxylic acid metabolism (ko01210), arginine biosynthesis (ko00220), and purine metabolism (ko00230) (Fig. 6E). This indicates a broad suppression in primary metabolism during the coculture.

## Discussion

This study reports for the first time that *Epicoccum nigrum* can be induced by *Mucor racemosus* to produce red pigments. *Mucor*-symbiotic systems are widely applied in traditional fermented food production. Sufu derived from the cofermentation with *M. racemosus* and *Rhizopus oryzae* demonstrated significantly higher key enzyme activities, biochemical quality, and textural (Wang et al. 2018). The coculture of *M. indicus* and *R. oryzae* enhanced key flavor compounds in Chinese turbid rice wine, and improved aroma intensity, taste harmony, and mouthfeel persistence and fullness (Xiang et al. 2019). It is acceptable that *E*. *nigrum* is the real producer of red pigments and *M. racemosus* as the inducer. Studies have shown that *E*. *nigrum* produces multiple secondary metabolites and pigments, including yellow and orange compounds such as carotenoids, azaphilones and epicolactone (Gribanovski-Sassu and Foppen 1967; Harwoko et al. 2020; Li et al. 2017, 2019; Shen et al. 2024). Besides, red pigments were rarely reported in *E*. *nigrum*. Rhodoxanthin and torularhodin were wine-red pigments produced by *E. nigrum*, but both were fat-soluble (Gribanovski-Sassu and Foppen 1967). Notably, the red pigments induced in our study are water-soluble, indicating the difference of *Mucor*-induced *Epicoccum* red pigments.

Although insufficient purified compound for NMR hindered absolute structural determination, preliminary analysis combining MS/MS data with SIRIUS suggested that the red pigments were putative anthracycline-like compounds. Similar to anthracyclines doxorubicin and epirubicin (Menna et al. 2006), compounds (1) and (4) share a tetracyclic framework. Anthracyclines are derived from the anthraquinone core scaffold, covalently linked to an aminosugar group and a tetrahydrofuran (THF) ring functional group, with their natural biosynthesis originating from Type II polyketide synthase (PKS) gene clusters (Lomovskaya et al. 1999). Notably, the fragments of these pigments appeared to show different side chains from classical anthracyclines, particularly in the tetracyclic core and the presence of phenylacetic acid moieties.

Based on the transcriptomic analysis and RT-qPCR results, genome mining proposed that a dual-PKS (*SPDX61809177* and *SPDX61809178*) cluster might be responsible for their biosynthesis (Fig. 4), which waiting to be confirmed in the future. *SPDX61809177* encodes a HR-PKS, whose modular architecture comprising KS/AT/DH/MT/ER/KR/ACP domains can be iteratively utilized to catalyze multiple elongation cycles. *SPDX61809178* encodes a NR-PKS containing SAT/KS/AT/ACP/MT/TD domains and exhibits high homology to MpPKS5 from *Monascus ruber* NRRL 1597, a polyketide synthase located within the azaphilone biosynthesis gene cluster (Balakrishnan et al. 2014). Similar dual-PKS organization commonly occurs in fungi (Zabala et al. 2012) and functions either in a sequential or in a convergent manner to produce the polyketide products, including hypothemycin (Reeves et al. 2008; Zhou et al. 2010), asperfuranone (Chiang et al. 2009), lovastatin (Ma et al. 2009), chaetoviridins/chaetomugilins, asperpyranones (Williams et al. 2021), and sclerotiorin (Sousa et al. 2023).

Moreover, converse to the enrichment of red pigments, much more secondary metabolites in *E. nigrum* showed lower abundance when cocultured with *M*. *racemosu*s, indicating that *E. nigrum* was suppressed by *M*. *racemosu*s. Notably, the production of epipyrone was decreased in coculture when compared to *E*. *nigrum* SPDX618 monoculture. Epipyrone has been reported as one of the yellow compounds produced by *E. nigrum* (Lima et al. 2020). And epipyrone was reported to have various biological activities, including antifungal, antiviral and antiproliferative activity (Lee et al. 2024; Longobardi et al. 2026). The reduced production of epipyrone is consistent with the downregulation of *epn* gene expressions in the coculture. Epipyrone is known to be produced also by a polyketide pathway, and *epnA* encodes a HR-PKS as well (Fan et al. 2024; Lima et al. 2020). Epipyrones and the red pigments share precursors such as acetyl-CoA and malonyl-CoA, as well as cellular energy requirements. When the metabolic routing turns into red pigment production, the biosynthesis of epipyrone gets blocked in *E*. *nigrum*. Although the ecological function of those red pigments is still unclear, reduced epipyrone production that contains antifungal activity places *E*. *nigrum* at a competitive disadvantage, which is consistent with the above observations.

Besides, multi-omics analysis revealed the underlying physiological mechanisms of pigment induction during cocultivation. Alterations in phosphatidylethanolamine (PE) and prostaglandin levels suggest the activation of membrane-mediated hyphal recognition and GPCR signaling pathways. As the second most abundant glycerophospholipid in cell membranes, PE has been reported to be related to diverse cellular functions, including phosphatidylcholine biosynthesis, posttranslational modifications, energy metabolism, etc. (Calzada et al. 2016). And the abundance of PE could positively regulate autophagy (Rockenfeller et al. 2015). Besides, it is also known that fungal-derived prostaglandins modulate sporulation and secondary metabolism via G protein-coupled receptor (GPCR)-mediated signaling pathways (Tsitsigiannis and Keller 2007). Meanwhile, the elevated levels of methionine sulfoxide and nucleoside derivatives indicated accumulated intracellular oxidative stress (Davies 2025; Kim et al. 2017). These results indicated a membrane-mediated fungal-fungal hyphae interaction and competition occurred during coculture.

The broad suppression of the identified core metabolic pathways indicate a comprehensive inhibition of primary metabolism during coculture. Since intermediates of primary metabolites are the precursors of secondary metabolites, the downregulation of these metabolites and their pathways limits the precursor supply for secondary metabolite production. Consequently, metabolic resources appear to be strategically redirected toward the specific activation of the red pigment biosynthetic cluster, rather than being distributed across multiple secondary metabolic routes. This reflects a metabolic trade-off wherein the fungus prioritizes the production of a specialized defensive metabolite in response to interspecific competition, at the expense of broader secondary metabolic diversification. Such dynamic flux redistribution highlights how fungal cocultivation can rewire metabolic networks through substrate channeling and cross-pathway regulation, effectively unlocking otherwise silent biosynthetic potential.

## Conclusion

In conclusion, we report a coculture system containing *Epicoccum nigrum* SPDX618 and *Mucor racemosus* SPDX522 from vinegar mash that constitutively produces anthracycline-like red pigments. *En*S618 acts as the producer and harbors an upregulated dual-PKS gene cluster during coculture that might be responsible for red pigment biosynthesis, while *Mr*S522 performs as the elicitor. Although red pigment biosynthesis got activated, much more secondary metabolites and primary metabolites were downregulated during coculture. This study primarily serves as a fundamental exploratory investigation into coculture-triggered metabolic reprogramming in fungi, laying a foundation for further research on the application potential of this coculture system and natural pigments.

## Supplementary Information

Below is the link to the electronic supplementary material.

ESM 1 (3.32 MB DOCX)

## Data Availability

The genomic and transcriptomic sequencing data generated in this study are available in the National Center for Biotechnology Information (NCBI) under BioProject accession number PRJNA1401448. Other data and materials will be available on request.
