# Supplementary material for: A coculture system of Epicoccum nigrum with Mucor racemosus induces red pigment biosynthesis in vinegar mash
Source: Appl Microbiol Biotechnol. 2026 Jul 23;110:311. doi: 10.1007/s00253-026-13956-1 (PMC13680316; doi:10.1007/s00253-026-13956-1)
Supplement: Supplementary file 1 [file 253_2026_13956_MOESM1_ESM.docx]

**Supporting Information**

**A Coculture System of *Epicoccum nigrum* with *Mucor racemosus* Induces Novel Pigment Biosynthesis in** **Vinegar Mash**

Fengli Wang, Fangyuan Xu, Xinle Liang^*^, Wenjie Wang^*^

*College of Food Science and Biotechnology, Zhejiang Gongshang University, Hangzhou, Zhejiang 310018, PR. China*

*Corresponding authors.

E-mail addresses: [dbiot@mail.zjgsu.edu.cn](mailto:dbiot@mail.zjgsu.edu.cn) (X. Liang), [wenjiewang@mail.zjgsu.edu.cn](mailto:wenjiewang@mail.zjgsu.edu.cn) (W. Wang).

**Fig S1.** Pigment yield of single-factor experiment. (A) Carbon source. (B) Temperature. (C) Initial pH. (D) Rice concentration. (E) Rice grain size. (F) Inoculation amount.

**Fig S2.** UV spectra of compounds 1-4.

**Fig S3.** Positive (A) ion mode and negative (B) ion mode UHPLC-MS of compound 1.

**Fig S4.** Positive (A) and negative (B) ion mode UHPLC-MS, and MS/MS spectra in positive ionization(C) of compound 2.

**Fig S5.** Positive (A) and negative (B) ion mode UHPLC-MS, and MS/MS spectra in positive ionization(C) of compound 3.

**Fig S6.** Elucidation of MS/MS fragment ions of compound 3.

**Fig S7.** Positive (A) and negative (B) ion mode UHPLC-MS, and MS/MS spectra in positive ionization(C) of compound 4.

**Fig S8.** Elucidation of MS/MS fragment ions of compound 4.

**Fig S9.** KEGG pathway enrichment analysis of downregulated metabolites in coculture.

**Table S1 Primers used in this study**

| Gene name | primer sequences（5’- 3’） | bp |
| --- | --- | --- |
| SPDX61809177 | GGTACCATGAGCCGATGAGA | 248 |
|  | CTCTGCCGGCTTCGTATAGA |  |
| SPDX61809178 | ACGAAGAAGCACAGAAGGGA | 214 |
|  | TGGTGGTGGTGGGAAATAGG |  |
| 18S | TATACGCTAGTGGAGCTGGAA | 147 |
|  | GGGGAGGTAGTGACGAAAAAT |  |

**Table S2 Levels and codes of variables in Plackett-Burman design experiments**

| level | *X_1_*  Temperature(℃) | *X_2_*  Initial pH | *X_3_*  Rice Concentration(g/L) | *X_4_*  Rice Grain Size (mm) | *X_5_*  Inoculation Amount (%) | *X_6_*  Fermentation Time (h) |
| --- | --- | --- | --- | --- | --- | --- |
| -1 | 20 | 4 | 30 | ＜ 0.18 mm | 5 | 72 |
| 1 | 28 | 10 | 70 | 5 mm | 10 | 108 |

**Table S3 The results of Plackett-Burman design test**

| Run | *X_1_* | *X_2_* | *X_3_* | *X_4_* | *X_5_* | *X_6_* | Y（U/g） |
| --- | --- | --- | --- | --- | --- | --- | --- |
| 1 | 1 | -1 | 1 | -1 | -1 | -1 | 49.9 |
| 2 | 1 | -1 | -1 | -1 | 1 | 1 | 78.8 |
| 3 | 1 | 1 | -1 | 1 | 1 | -1 | 82.8 |
| 4 | -1 | 1 | 1 | 1 | -1 | 1 | 94.7 |
| 5 | -1 | -1 | -1 | 1 | 1 | 1 | 138.6 |
| 6 | 1 | -1 | 1 | 1 | -1 | 1 | 77.8 |
| 7 | -1 | 1 | -1 | -1 | -1 | 1 | 155.1 |
| 8 | 1 | 1 | -1 | 1 | -1 | -1 | 54.8 |
| 9 | -1 | -1 | 1 | 1 | 1 | -1 | 65.5 |
| 10 | -1 | 1 | 1 | -1 | 1 | -1 | 42.4 |
| 11 | -1 | -1 | -1 | -1 | -1 | -1 | 60.6 |
| 12 | 1 | 1 | 1 | -1 | 1 | 1 | 40.5 |

**Table S4 Analysis of variance for Plackett-Burman**

| Source | df | SS | MS | *F* | *P* |
| --- | --- | --- | --- | --- | --- |
| Model | 7 | 25773.7 | 3682.0 | 7.60 | 0.008 |
| Linear | 6 | 10986.5 | 1831.1 | 3.78 | 0.053 |
| *X_1_* | 1 | 2472.5 | 2472.5 | 5.10 | 0.058 |
| *X_2_* | 1 | 0.1 | 0.1 | 0.00 | 0.991 |
| *X_3_* | 1 | 3331.0 | 3331.0 | 6.88 | 0.034 |
| *X_4_* | 1 | 628.9 | 628.9 | 1.30 | 0.292 |
| *X_5_* | 1 | 163.8 | 163.8 | 0.34 | 0.579 |
| *X_6_* | 1 | 4390.3 | 4390.3 | 9.06 | 0.020 |
| curvature | 1 | 14787.2 | 14787.2 | 30.53 | 0.001 |
| Error‌ | 7 | 3390.3 | 484.3 |  |  |
| ‌Lack of Fit‌ | 5 | 3390.3 | 678.1 | * | * |
| Pure Error | 2 | 0.0 | 0.0 |  |  |

Note：*Y* = 78.49 - 14.35 *X_1_* - 0.08 *X_2_* - 16.66 *X_3_* + 7.24 *X_4_* - 3.69 *X_5_* + 19.13 *X_6_* - 78.5 Ct Pt

**Table S5 The results of Box-Behnken design test**

| Run | *X_1_*/*A*  (Temperature) | *X_3_*/*B*  (Rice Concentration) | *X_6_*/*C*  (Fermentation Time) | Y(U/g) |
| --- | --- | --- | --- | --- |
| 1 | -1 | 0 | 1 | 141.8 |
| 2 | 0 | 0 | 0 | 161.4 |
| 3 | 0 | 0 | 0 | 158.5 |
| 4 | -1 | 1 | 0 | 145.6 |
| 5 | -1 | 0 | -1 | 130.5 |
| 6 | 0 | -1 | -1 | 126.5 |
| 7 | -1 | -1 | 0 | 124.5 |
| 8 | 0 | 1 | 1 | 146.8 |
| 9 | 0 | 1 | -1 | 132.7 |
| 10 | 1 | 0 | 1 | 120.5 |
| 11 | 1 | 1 | 0 | 131.2 |
| 12 | 1 | 0 | -1 | 127.2 |
| 13 | 0 | -1 | 1 | 118.5 |
| 14 | 0 | 0 | 0 | 165.5 |
| 15 | 1 | -1 | 0 | 125.5 |

**Table S6 ANOVA analysis for regression equation**

| Source | df | SS | MS | *F* | *P* |
| --- | --- | --- | --- | --- | --- |
| Model | 9 | 3218.26 | 357.585 | 37.16 | 0.000 |
| Linear | 3 | 664.52 | 221.508 | 23.02 | 0.002 |
| *A* | 1 | 180.50 | 180.500 | 18.76 | 0.007 |
| *B* | 1 | 469.71 | 469.711 | 48.81 | 0.001 |
| *C* | 1 | 14.31 | 14.311 | 1.49 | 0.277 |
| Square | 3 | 2291.35 | 763.783 | 79.37 | 0.000 |
| *A*^2^ | 1 | 900.00 | 900.001 | 93.53 | 0.000 |
| *B*^2^ | 1 | 774.97 | 774.970 | 80.54 | 0.000 |
| *C*^2^ | 1 | 967.51 | 967.514 | 100.55 | 0.000 |
| Two-way interaction | 3 | 262.39 | 87.464 | 9.09 | 0.018 |
| *AB* | 1 | 59.29 | 59.290 | 6.16 | 0.056 |
| *AC* | 1 | 81.00 | 81.000 | 8.42 | 0.034 |
| *BC* | 1 | 122.10 | 122.103 | 12.69 | 0.016 |
| Error‌ | 5 | 48.11 | 9.623 |  |  |
| ‌Lack of Fit‌ | 3 | 23.37 | 7.791 | 0.63 | 0.661 |
| Pure Error | 2 | 24.74 | 12.370 |  |  |
| Total | 14 | 3266.38 |  |  |  |

Note:*Y* = -4422 + 215.5 *A* + 35.11 *B* + 162.0 *C* - 3.903 *A*^2^ - 0.1789 *B*^2^ - 16.19 *C*^2^ - 0.2139 *AB*- 2.250 *AC* + 0.614 *BC*
